# The influence of the pollination compatibility type on the pistil *S-RNase* expression in European pear (*Pyrus communis*)

**DOI:** 10.3389/fgene.2024.1360332

**Published:** 2024-04-09

**Authors:** Hanne Claessen, Han Palmers, Wannes Keulemans, Bram Van de Poel, Nico De Storme

**Affiliations:** ^1^ Laboratory for Plant Genetics and Crop Improvement, Division of Crop Biotechnics, Department of Biosystems, University of Leuven, Leuven, Belgium; ^2^ KU Leuven Plant Institute (LPI), University of Leuven, Leuven, Belgium; ^3^ Laboratory for Molecular Plant Hormone Physiology, Division of Crop Biotechnics, Department of Biosystems, University of Leuven, Leuven, Belgium

**Keywords:** pear (*Pyrus communis*), gametophytic self-incompatibility, *S-RNase*, pollen-style interactions, allele-specific expression

## Abstract

The *S-RNase* gene plays an essential role in the gametophytic self-incompatibility (GSI) system of *Pyrus*. It codes for the stylar-expressed S-RNase protein which inhibits the growth of incompatible pollen tubes through cytotoxicity and the induction of programmed cell death in the pollen tube. While research on the *Pyrus* GSI system has primarily focused on the *S-RNase* gene, there is still a lack of insight into its spatiotemporal expression profile and the factors that regulate it. Previous studies have suggested that *S-RNase* expression in the style is influenced by pollination and is dependent on the compatibility type. We here continue on this basic hypothesis by analyzing the spatiotemporal expression of the *S-RNase* alleles in *Pyrus communis* “Conference” styles in response to different types of pollination; namely, upon full- and semi-compatible pollination and upon incompatible selfing. The results revealed that temporal dynamics of *S-RNase* expression are influenced by the pollen’s compatibility type, indicating the presence of a signaling mechanism between pollen and style to control *S-RNase* production during pollen tube growth. In our experiment, *S-RNase* expression continuously decreased after cross-pollination and in the unpollinated control. However, after a fully incompatible pollination, *S-RNase* expression remained constant. Finally, semi-compatible pollination showed a initially constant *S-RNase* expression for both alleles followed by a strong decrease in expression. Based on these results and previous findings, we propose a regulatory mechanism to explain the effect of pollination and the associated compatibility type on *S-RNase* expression in the style. This proposed mechanism could be used as a starting point for future research.

## 1 Introduction

Upon germination of the pear (*Pyrus communis*) pollen on the stigma, the pollen tube passes through the specialized style transmitting tract (TT) which comprises a dense extracellular matrix (ECM) containing factors that can promote or hinder pollen tube growth. These factors are established by the continuous signal exchanges occurring between the growing pollen tubes and the style. Some examples of such signals include the chemo-attractants produced by the ovule that serve as guidance cues for the pollen tube, alterations in the metabolic status of the stylar transmitting tract in response to pollen tube germination and growth, and pollination-induced pistil aging and ovule maturation (Dresselhaus and Franklin-tong, 2013; Lora et al., 2016; Joly et al., 2019; Mandrone et al., 2019). Self-incompatibility is another important type of pollen-style interaction. In pear (*Pyrus* genus), inhibition of self-fertilization is regulated at the genetic level by the *S-RNase-*dependent gametophytic self-incompatibility (GSI) system, which is determined by a single locus; the S-locus. This locus comprises the female *S-RNase* gene (Sassa et al., 1992) and multiple male *S-locus F-box brother* (*SFBB*) genes (Sassa et al., 2007) that are tightly linked in one linkage block. The S-RNase protein is the female-specific GSI component and is expressed solely in the pistil. The S-RNase is a member of the RNase T2 family, but while the RNase activity of this protein is known to be required for the pistil S function in Solanaceae (Huang et al., 1994), it remains unclear whether only RNA degradation triggers pollen tube growth inhibition since the S-RNases of pear and apple were also shown to interact with other targets such as actin (Liu et al., 2007), phospholipase C (Qu et al., 2017), and pyrophosphatase (Li et al., 2018). Upon pollen germination, the stylar S-RNase proteins enter the cytosol of each growing pollen tube non-selectively (Luu et al., 2000; Goldraij et al., 2006; Hui et al., 2014), however, they only inhibit the growth of incompatible pollen tubes (De Franceschi et al., 2012). In contrast, the *SFBB* genes, as male-specific GSI component, are expressed in the growing pollen tube where they encode different F-box proteins that target specific S-RNases that enter the pollen tube from the transmitting tract. The molecular mechanism of GSI regulation by S-RNases and SFBBs is described in the non-self-recognition model. In this model, it is proposed that growing pollen tubes express many different SFBBs that specifically interact with invading non-self-S-RNases (Kubo et al., 2010), whereby they act as a subunit of the SCF complex to ubiquitinate and mark these S-RNases for degradation by the 26S proteasome, hence ensuring continued pollen tube growth in case of a compatible interaction (Hua et al., 2008). In case of a non-compatible pollination (e.g, selfing), the SFFBs in the pollen tube cannot interact with the invading self-type S-RNases and mark them for degradation, so that these S-RNases remain present and can exert their cytotoxic function to inhibit pollen tube growth. This non-self-recognition model of GSI was first proposed in Petunia (Kubo et al., 2010) after which it was also adopted for apple and pear based on the identification of multiple *SFBB* genes at the S-locus of Maleae (De Franceschi et al., 2011), the observed competitive interaction in heteroallelic pollen (Qi et al., 2011; De Franceschi et al., 2012), and the characterization of the self-compatible mutant S^4sm^ haplotype (Okada et al., 2008).

The *S-RNase* gene is believed to be expressed in the cells of the stylar transmitting tract and released into the extracellular matrix (Sassa et al., 1993; McClure et al., 2011). However, there is only limited information available on the exact spatiotemporal expression profile of this gene and the factors that regulate or influence its expression. Studies on different species with *S-RNase*-dependent GSI have suggested that pollination may influence *S-RNase* expression in the style with effects depending on the compatibility type. For instance, *Solanum chacoense* was found to exhibit a 60% decrease in *S-RNase* mRNA levels in the styles 24 h after cross-pollination, compared to a more moderate 25% decrease after selfing (Liu et al., 2009). Based on these findings, the authors proposed a positive feedback mechanism in which compatible pollen tubes simultaneously degrade the S-RNase protein in their cytoplasm and send out a signal to reduce *S-RNase* expression, hence reinforcing the compatible reaction (Liu et al., 2009). A decreased *S-RNase* expression after cross-pollination and higher expression after self-pollination was also found in lemon, a member of the Rutaceae family which was recently also found to harbor a similar type of S-RNase-dependent GSI (Honsho, 2023). Expression analysis of the *S3-RNase* in lemon styles showed ten times higher expression after self-pollination compared to cross-pollination 20 h after pollination (Li et al., 2022). In the *Pyrus bretschneideri* cultivar “Dangshan,” the expression of both *S-RNase* alleles (*Pb*S_7_ and *Pb*S_34_) also decreased after cross-pollination and this occurred faster and more pronounced as compared to no pollination or self-pollination (Shi et al., 2017). These results indicate that the compatibility type of pollination affects *S-RNase* expression in the style and suggests the presence of a yet unknown signaling mechanism that affects the molecular control of the GSI system. However, up till now, experimental data in pear or related species, such as apple, is very limited and a possible mechanism has as of yet not been proposed. While the GSI system in Solanaceae and Rosaceae share many similarities, there are also important differences, such as the involvement of non-S-locus factors HT-B and 120K in Solanaceae (Goldraij et al., 2006), which have not been observed in Maleae. Therefore, it cannot simply be assumed that the positive feedback mechanism proposed in *S. chacoense* by Liu et al. (2009) also applies to pear.

In this study, we used the *P. communis* cultivar “Conference” as a model to monitor allele-specific *S-RNase* expression in unpollinated and pollinated flowers at different time points following (no) pollination. *S-RNase* expression was assessed in two regions of the style (upper and lower) and the impact of the three compatibility types was assessed: compatible, incompatible, and semi-compatible pollination. Based on the obtained results and information from previous studies, we propose a mechanistic model for feedback regulation of *S-RNase* expression depending on the pollination compatibility type in *P. communis* that could serve as a valuable starting point to further elucidate the molecular cues and pathways underpinning the intricate GSI system in pear.

## 2 Materials and methods

### 2.1 Plant material

The plant material used for this study included mature potted trees of *P. communis* cultivars “Conference,” “Légipont” and “Bartlett” (“Williams Bon Chrétien”). In order to have available pollen donors for performing the different types of *in situ* pollination, one tree of each pear variety was brought into the greenhouse approximately 2 weeks before the expected flowering time and was forced into flowering under controlled conditions. Light intensity in the greenhouse was maintained at >240 W/m^2^ between 7:00–19:00 (using supplemental Son-T lights) and the temperature was held within the range of 15°C–27°C, at an average of 21.5°C. For all three cultivars, approximately 100 flowers were harvested at the balloon stage for pollen collection. Unopened anthers were collected using forceps into a small Petri dish and air dried for 48 h until opening to release mature pollen grains. Dried pollen grains were stored until pollination at room temperature in air-tight containers containing silica gel. Pollinations were performed on flowers of the cultivar “Conference.” Five adult potted trees of cultivar “Conference” were brought into the greenhouse at the onset of flowering and were further maintained under the same conditions outlined above to minimize environmental effects during the experiment.

### 2.2 Pollinations and style tissue sampling

**FIGURE 1:**
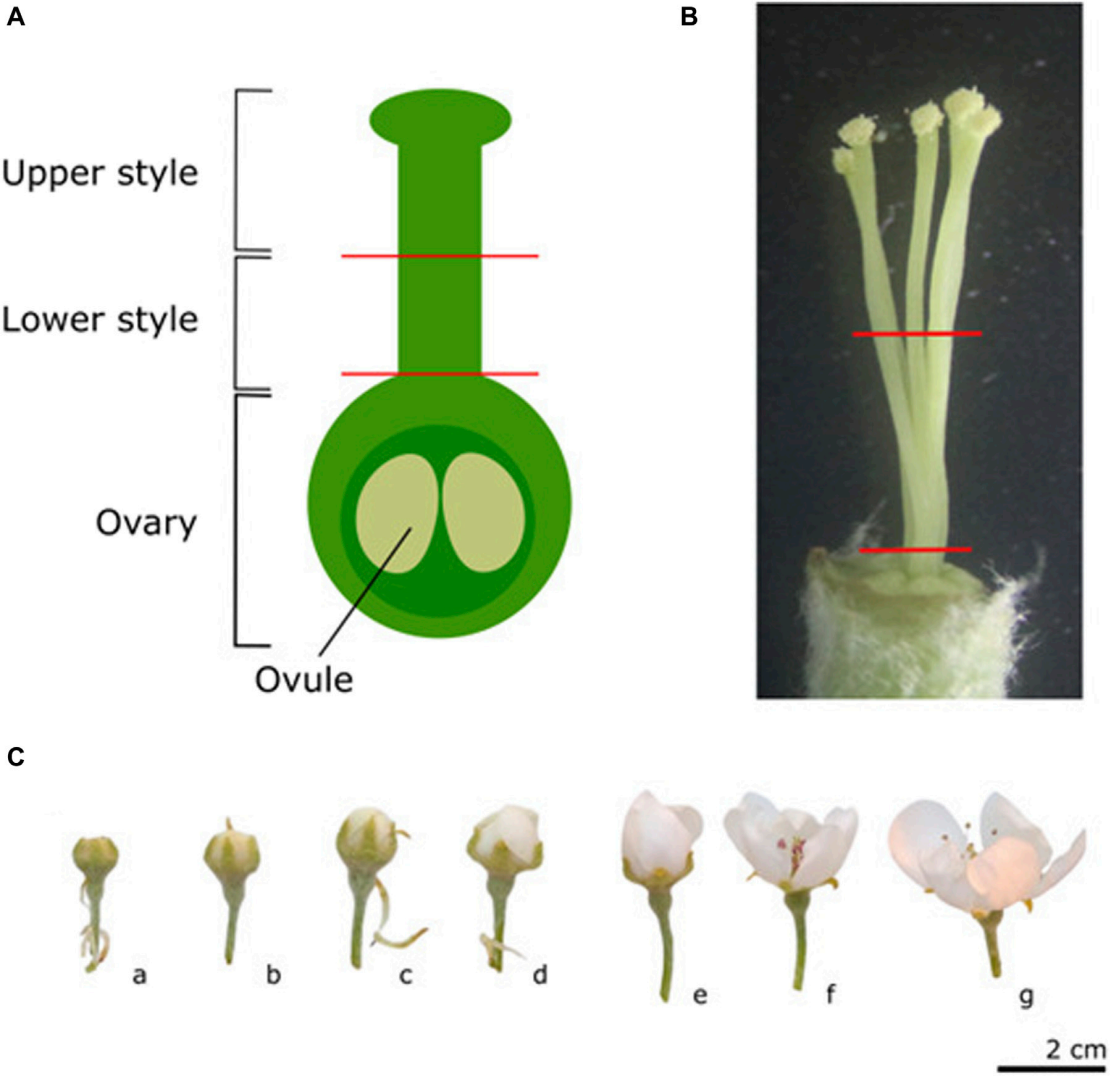
Schematic representation of the different parts of the pistil used for sampling **(A)**, and a reference picture of the five pear styles of cultivar “Conference” to indicate the section sites used for tissue sampling **(B)**. Representative images of the different phenological stages of flower development **(C)**. a) Green bud stage: single flowers are visible but still closed. b-c) White/pink bud stage: petals start to appear and petal color is cultivar-dependent during this stage, the styles may still be very short. d) Start of the balloon stage: flowers are still closed and stamens are still tightly packed around the style. e) Balloon stage: petals are still closed, but can easily be moved aside. f) Anthesis: anthers are pink and closed but the petals have opened. In this stage, flowers can no longer be considered unpollinated since insects carrying pollen from nearby trees may have visited the flower; g) Open flower: anthers have dehisced.

**FIGURE 2:**
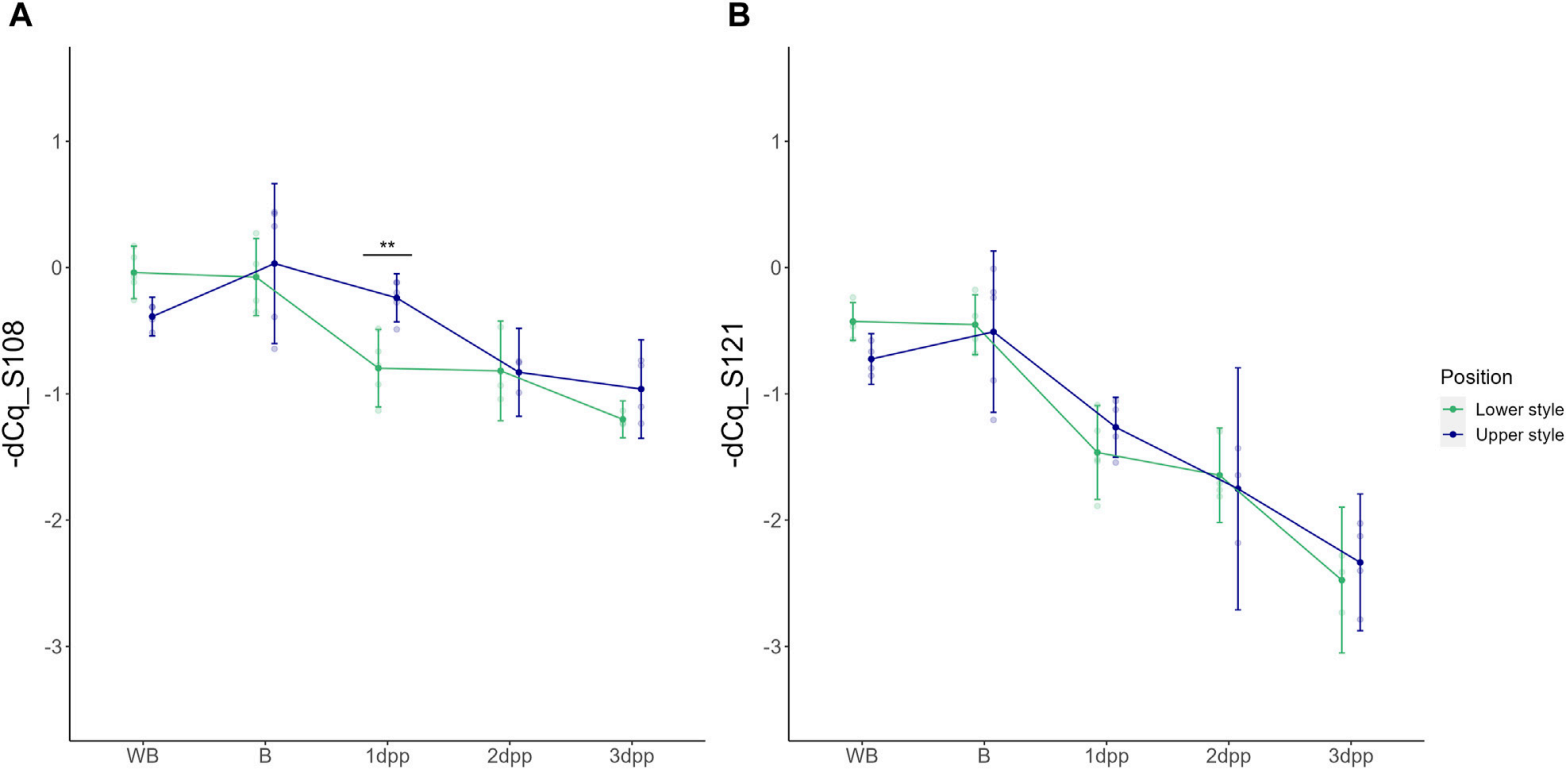
Temporal dynamics of the *Pc*S_108_ **(A)** and *Pc*S_121_ **(B)** *S-RNase* expression levels denoted as -dCq values in both the upper and lower half of the style across five time points throughout development of an unpollinated style of the cultivar “Conference.” Data points represent mean values, error bars represent 95% confidence intervals. Asterisks mark the time points at which there is a significant difference between *S-RNase* allele expression in the upper versus the lower part of the style (*p* < 0.05) with *: *p* < 0.05, **: *p* < 0.01 and ***: *p* < 0.001.

Controlled pollinations were all performed using “Conference” as pollen acceptor. On each of the five “Conference” trees, four individual branches were labeled, each to receive one of the four possible pollination types. Flowers on these branches were pruned and only flowers in the same developmental stage (balloon stage) and having the same visually assessed fitness (>6 flowers in the cluster, uniform flower size, and no visible signs of disease or insect damage) were maintained for controlled pollination. The different pollination types included no pollination (UP), self-pollination (Self), cross-pollination (Cross), and semi-compatible pollination (Semi). For the three pollination treatments (Self, Semi, and Cross), pollen was collected before the start of the experiment from forced “Conference” (Self, *Pc*S_108_-*Pc*S_121_), “Légipont” (Semi, *Pc*S_102_-*Pc*S_108_) and “Bartlett” (Cross, *Pc*S_101_-*Pc*S_102_) trees, respectively. Flowers of the “Conference” pollen acceptor trees were emasculated (except for the Self-treatment) and hand-pollinated at the balloon stage by manually applying the dried pollen to the stigmas using thin, soft bristle paint brushes. New, individually packaged brushes were used for each pollination type to avoid cross-contamination. Following emasculation and pollination, flowers were immediately bagged. Sampling of “Conference” styles was performed at different time points both before and after pollen application; including the white bud stage (Figure 1C, c), balloon stage (Figure 1C, e), and 1, 2, and 3 days after pollination (dpp) which was performed at balloon stage. White bud and balloon stages were sampled before pollination and were included to assess *S-RNase* expression in the early developmental stages before anthesis and without pollination. For each time point and pollination type a total number of 30 flowers was harvested. Flowers were sampled randomly from the five “Conference” trees with each sampling moment including flowers from at least three different trees. From each flower, the styles were dissected from the pistil and cut at two different heights using sterilized surgical scissors (Figures 1A, B). Styles were cut halfway, and the upper and lower halves of the styles were collected separately in an Eppendorf tube. In order to have sufficient material for mRNA extraction, isolated style parts from six flowers were pooled into one sample. This process was repeated five times for each pollination type and sampling time point to consistently obtain five biological replicates per treatment. Samples were immediately flash-frozen and stored at −80°C to minimize RNA degradation.

### 2.3 Validation of the S-genotype by PCR amplicon sequencing

S-genotypes of the “Conference,” “Bartlett” and “Légipont” trees were validated before the start of the experiment to ensure the desired compatibility relations. DNA was extracted from young leaves using the Nucleospin^®^ plant II DNA extraction kit (Macherey-Nagel) following the manufacturer’s guidelines. DNA concentration and quality were checked using a Nanodrop 2000 spectrophotometer (Thermo Scientific). Consensus and allele-specific primers (Supplementary Table S1) as described in Sanzol (2009) were used to amplify the *S-RNase* allele sequences based on the expected S-genotypes as derived from literature, being *Pc*S_108_-*Pc*S_121_ for “Conference” (Goldway et al., 2009), *Pc*S_102_-*Pc*S_108_ for “Légipont” (Quinet et al., 2014) and *Pc*S_101_-*Pc*S_102_ for “Bartlett” (Goldway et al., 2009). Multiple primers were used to obtain the full coding sequence of each S-allele. PCR-based S-genotyping using consensus primers was performed as described in Sanzol (2009). Allele-specific PCRs were performed in a T100TM Thermal Cycler (Bio-Rad) using a standard PCR program: hot-start of 2 min at 95°C; 35 cycles of 30 s at 95°C, 1 min at a primer-dependent annealing temperature (Supplementary Table S1) and 2 min at 72°C; followed by a final cycle of 10 min at 72°C. The PCR mix for each sample contained 50 ng of DNA, 5 µL of 10X DreamTaq buffer (Thermo Scientific), 0.2 mM of dNTP mixture, 0.5 µM of each primer, and 1.25U DreamTaq DNA polymerase (Thermo Scientific), supplemented with dH_2_O to reach a final volume of 50 µL. The resulting PCR products were separated by gel electrophoresis using 1% agarose gels containing 10 mg/mL UltraPureTM Ethidium Bromide (Invitrogen) after which they were visualized using the Gel Doc EZ documentation system (Bio-Rad). PCR amplicon bands with the correct fragment length were cut out of the gel, purified using the GeneJet PCR purification kit (Thermo Scientific), and subsequently Sanger sequenced in both directions using corresponding PCR primers (LGC Genomics, Hoddesdon, United Kingdom). Resulting amplicon sequences were trimmed and corresponding forward and reverse fragments of the different overlapping S-allele parts were aligned using Geneious^®^ v11.1.5 software to obtain a complete consensus gDNA sequence of each S-allele. These PCR amplicon sequences were then subjected to a nucleotide BLAST analysis against the NCBI nucleotide database (http://blast.ncbi.nlm.nih.gov) to determine the corresponding *S-RNase* allele.

### 2.4 RNA extraction and cDNA synthesis

Total RNA was extracted from the collected style fragments using the Nucleospin^®^ RNA Plant and Fungi kit (Macharey-Nagel) according to the manufacturer’s instructions. Frozen samples were prepared for extraction by adding the lysis buffer and by thoroughly crushing the sample with a decontaminated pestle. Following RNA extraction, putative DNA contamination was removed using the DNA-free™ DNA removal kit (Thermo Scientific) according to the manufacturer’s protocol. The integrity of resulting RNA samples was checked by agarose gel staining (1.5% gel stained with ethidium bromide) and RNA content was quantified using a Nanodrop 2000 spectrophotometer (Thermo Fisher Scientific) by measuring the absorption at 260 nm. For each sample, 455 ng of purified RNA was reverse transcribed into complementary DNA (cDNA) using the SuperScript™ Reverse Transcriptase kit (Thermo Scientific) according to the manufacturer’s protocol after which the resulting cDNA was stored at −20°C.

### 2.5 Quantitative RT-PCR

To specifically detect and quantify the expression of the *Pc*S_108_ and *Pc*S_121_ alleles in “Conference” pistils, new primer pairs were designed for each allele using the NCBI primer blast tool (Supplementary Table S1). The RT-qPCR reaction mixtures contained 4 µL cDNA, 0.4 µM of each primer, and 1X SYBR Green master mix (BioRad), and were supplemented with dH_2_O to reach a final volume of 20 µL. The RT-qPCR program consisted of 40 cycles of 15 s at 95°C and 15 s at 60°C, followed by melting curve analysis from 65°C to 95°C with 0.5°C increments at 5 s/step. The qPCR reaction was performed in the CFX-96 Touch Real-Time PCR detection system (BioRad). At the end of each qPCR run, a high-resolution melting curve analysis was performed to check product specificity. The specificity of the primer pairs was additionally determined by Sanger sequencing (LGC Genomics) of the purified PCR product (GeneJet PCR Purification kit) for three independent samples of each primer pair. *S-RNase* allele expression measurements were normalized against the average expression of two reference genes, namely, *Histone H3* (XM_009367550.2) and *EF1α* (AY338250) (Liu et al., 2018). Primer specifications for both reference genes are listed in Supplementary Table S1. A calibration curve was run in triplex in each run consisting of a two-fold serial dilution ranging up to 2^–5^ of a mixed cDNA sample of all samples in the experiment. The qPCR reactions were performed over different plates in an “all samples” approach where only one gene was tested per plate. To correct for any offset between plates, three inter-run calibrators (IRC) were measured per plate. Raw Cq values for all tested genes were corrected for plate-specific primer efficiency, as calculated from the calibration curves using the method described in Ganger et al. (2017), and normalized across different plates using the IRCs. The dCq value for each sample was calculated by subtracting the mean Cq value of the reference genes from the corresponding raw Cq value of the sample.

### 2.6 Statistics and data analysis

All statistical tests were performed in R (version R 4.2.0). Before any statistical processing, normality of the subpopulations in the data was determined using the Shapiro-Wilk test. Similarly, heteroscedasticity was evaluated through the Bartlett’s test of homogeneity of variances. Differences in the spatiotemporal expression of the two S-alleles across all time points in the unpollinated flowers were evaluated separately for each *S-RNase* allele using a two-way ANOVA including the fixed factors “time” and “position” and their interaction effects. The significant interaction effect between time and position for *Pc*S_108_ was further investigated via a simple main effects analysis using the Phia package (De Rosario-Martínez, 2024). In addition, a three-way ANOVA was performed to analyze the effect of the pollination treatment (i.e., compatibility type), stylar position, and time on the expression of each *S-RNase* allele. Significant interaction effects were further investigated via a simple main effects analysis using the Phia package (De Rosario-Martínez, 2024). Multiple comparisons within the simple-effect analysis and evaluation of significance of differences were performed using Tukey’s HSD *post hoc* test with a significance level of 0.05. Results were plotted below as mean expression values and 95% confidence intervals.

## 3 Results

### 3.1 Validation of the S-genotype of the experimental pear cultivars

The PCR using consensus primers PyComC1F and PyComC5R yielded two bands close together at 680 bp (*Pc*S_108_) and 661 bp (*Pc*S_121_) for “Conference,” two bands at 1,306 bp (*Pc*S_101_) and 1,723 bp (*Pc*S_102_) for “Bartlett,” and two bands at the expected sizes of 1,723 bp (*Pc*S_102_) and 680 bp (*Pc*S_108_) for “Légipont” (Supplementary Figure S1), strongly matching the expected S-genotypes of the three cultivars (Sanzol, 2009). Sanger sequencing of the purified PCR products using allele-specific primers followed by Blast analysis of the resulting nucleotide sequences confirmed the identities of the four expected *S-RNase* alleles. An overview of the obtained consensus sequences and their alignment to their reference sequence is included in Supplementary Figures S2–S5.

### 3.2 Spatio-temporal expression dynamics of both *S-RNase* alleles in unpollinated pistils

As a first analysis, the basal spatio-temporal expression profile of the two “Conference” S-alleles was evaluated across all time points in unpollinated styles (UP treatment). Under these conditions, both *S-RNase* alleles show highest expression before anthesis (i.e., B and WB stages) after which their relative transcript level gradually decreases in the subsequent days. In the case of unpollinated styles the abbreviations 1dpp, 2dpp and 3dpp refer to days after balloon stage rather than days after pollination since no pollination was performed in this case. A two-way ANOVA statistical test hereby showed a significant main effect of time for both *Pc*S_108_ [F (4,33) = (23,237), *p* = 3,327e-09] and *Pc*S_121_ [F (4,33) = (62,650), *p* = 6,003e-15] and a significant interaction between time and position for *Pc*S_108_ [F (4,33) = (3,875), *p* = 1,092e-02]. This indicates that the temporal expression pattern of the *Pc*S_108_ allele depends on the position in the style, and thus differs between the higher and lower part of the style. A significant difference in *Pc*S_108_ expression between the upper and lower part of the style was measured at 1 dpp (*p* = 8,115e-03), however, not at the other time points. In contrast, no significant differences in spatial expression were observed for *Pc*S_121_ (Figure 2).

### 3.3 *S-RNase* expression profile in the pistil differs depending on the pollination type

The effect of pollination events with different compatibility types (compatible, incompatible, and semi-compatible) on the temporal expression dynamics of *PcS*_*108*_ and *PcS*_*121*_ was assessed at three successive time points following pollination for both the upper and lower region of the style.

**FIGURE 3:**
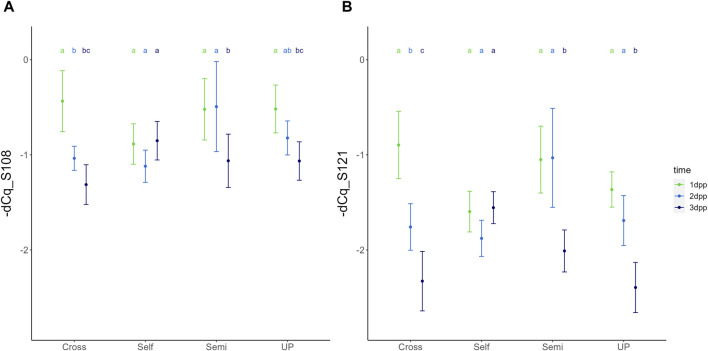
Temporal dynamics of the expression of *Pc*S_108_ **(A)** and *Pc*S_121_ **(B)** *S-RNase* alleles in the styles of the cultivar “Conference” upon different types of pollination treatments, including unpollinated (UP), cross-pollination (Cross), selfing (Self) and semi-compatible pollination (Semi). Data points represent mean values, error bars represent 95% confidence intervals. Letters above the data points indicate significant differences between time points for specific pollination treatments as determined by the Tukey post-hoc test with a significance level of 5% (α = 0.05).

**FIGURE 4:**
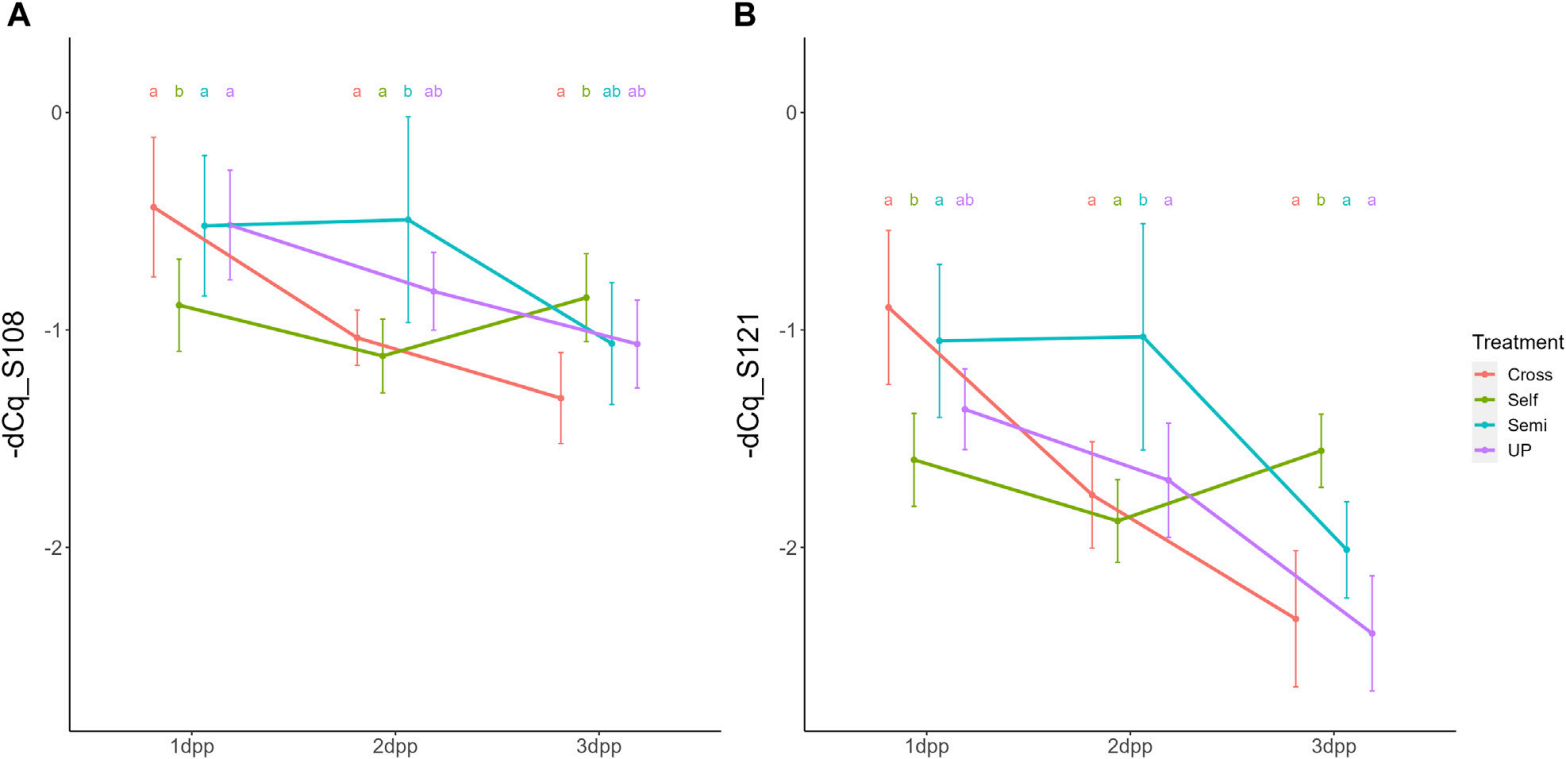
Temporal dynamics of the expression of *Pc*S_108_ **(A)** and *Pc*S_121_ **(B)** *S-RNase* alleles in the styles of the cultivar “Conference” upon different types of pollination treatments, including unpollinated (UP), cross-pollination (Cross), selfing (Self) and semi-compatible pollination (Semi). Data points represent mean values, error bars represent 95% confidence intervals. Letters above the data points indicate significant differences between treatments at a specific time point as determined by the Tukey post-hoc test with a significance level of 5% (α = 0.05).

Expression dynamics of both *S-RNase* alleles were highly similar for each type of pollination treatment, generally showing a gradual reduction in transcript levels upon pollination, though with marked differences in temporal dynamics between the different compatibility types (Figures 3, 4). For *Pc*S_108_, the three-way ANOVA revealed that all three fixed factors, i.e., pollination treatment, position in the style, and time have a significant effect on the relative expression level, with significant interactions between treatment and time [F (6,70) = (9,911), *p* = 6,743e-08], and treatment and position in the style [F (3,70) = (4,005), *p* = 1,086e-02]. Similarly, for *Pc*S_121_, significant main effects for all three independent factors were retrieved, as well as significant interactions between treatment and time [F (6,70) = (15,180), *p* = 4,771e-11] and treatment and position [F (3,70) = (4,549), *p* = 5,706e-03].

The significant interaction between treatment and time indicates that the temporal expression profile of both *S-RNase* alleles depends on the pollination treatment, with both *S-RNase* alleles responding highly similar for each of the compatibility type pollinations. Figure 3 shows the comparison of the *Pc*S_108_ (A) and *Pc*S_121_ (B) expression levels between time points for each pollination treatment. Reversely, Figure 4 shows the comparison of the *Pc*S_108_ (A) and *Pc*S_121_ (B) expression levels between pollination treatments for each time point. In the complete absence of pollination (UP), expression of both *S-RNase* alleles progressively and significantly drops representing the baseline expression dynamics of each *S-RNase* allele (Figure 3). A similar progressive and significant reduction in *S-RNase* expression is observed for both alleles after cross-pollination (Cross). In contrast, following self-pollination, the expression of both *S-RNase* alleles shows a constant trend (Figure 3), after an initially lower expression at 1 dpp that is significant compared to the other treatments for both *S-RN*ase alleles (Figure 4). This constant *S-RNase* expression after self-pollination is reflected by a significantly higher expression of the two *S-RNase* alleles at 3 days following selfing as compared to cross-pollination (for *Pc*S_108_) and compared to all other treatments (for the *Pc*S_121_ allele) (Figure 4). These results suggest that whereas *S-RNase* expression keeps decreasing after full-compatible cross-pollination, i.e., mimicking the baseline expression occurring in the absence of pollination (UP), *S-RNase* expression is maintained after self-pollination. Strikingly, following a semi-compatible pollination, the expression profile of both *S-RNase* alleles exhibits a constant level until 2 days after pollination followed by a delayed drop in expression that only occurs at 3 dpp (Figure 3). This maintained expression is reflected by a significantly higher transcript level of both *S-RNase* alleles at 2 dpp as compared to the other pollination treatments (Figure 4).

**FIGURE 5:**
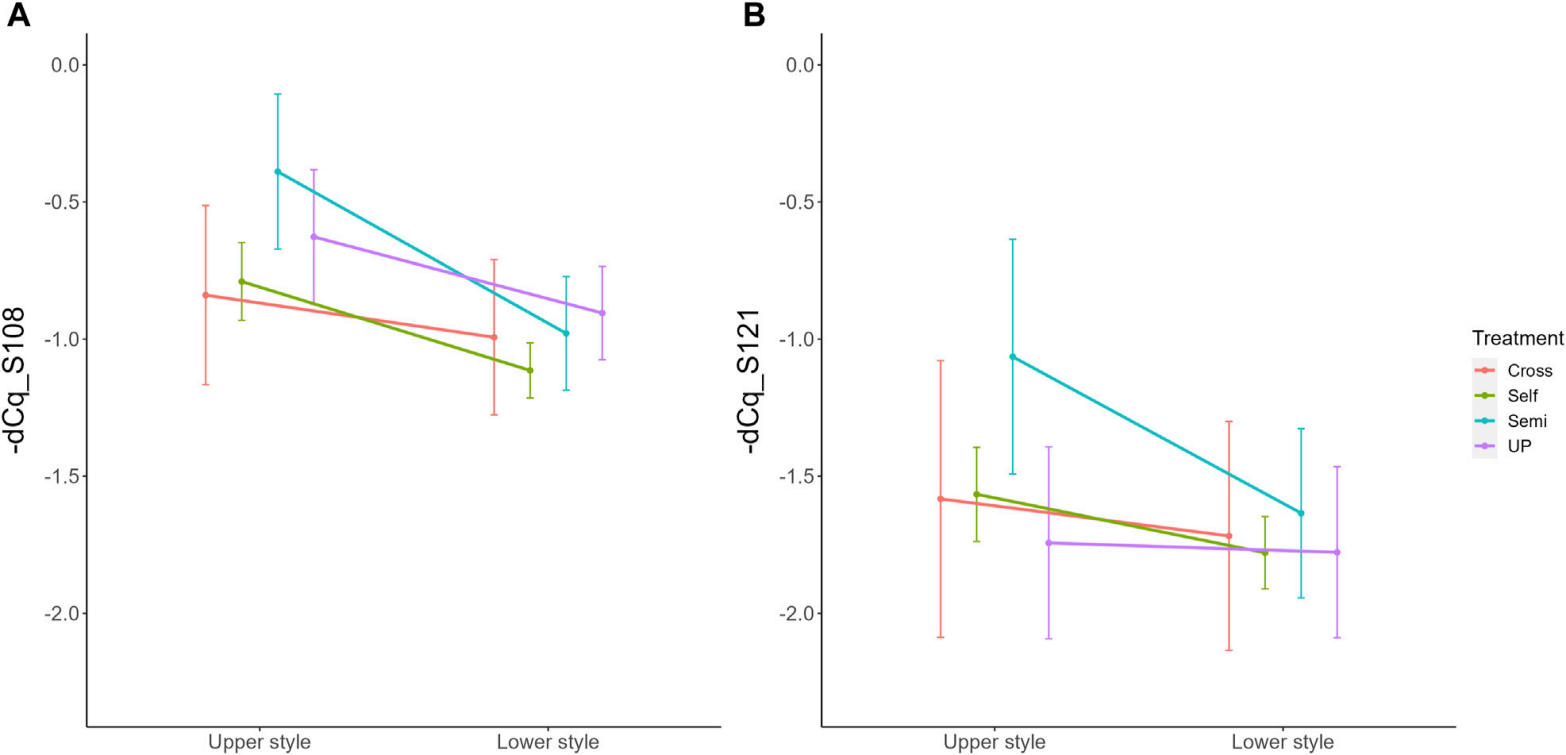
Interaction plots for the significant interaction effect between pollination treatment and position for both *Pc*S_108_ **(A)** and *Pc*S_121_ **(B)** *S-RNase* expression levels in styles of the cultivar “Conference”. Data points represent mean values, error bars represent 95% confidence intervals. Letters above the data points indicate significant differences between treatments over all time points per style region as determined by the Tukey post-hoc test with a significance level of 5% (α = 0.05).

Parallel to the interaction between pollination treatment and time, there was also a significant interaction effect between treatment and position within the style (i.e., lower versus upper part) for both *S-RNase* alleles. The interaction plot between both variables is provided in Figure 5 and shows that the difference in *S-RNase* expression between treatments depends on the position in the style and that the effect of the pollination treatment is largest in the upper style region. Significant differences between treatments, taking together all time points, were only observed in the upper region of the style with the semi-compatible pollination being significantly different from the cross- and self-pollination treatment. This difference was not observed in the lower style. This result suggests that the delayed drop in expression after semi-compatible pollination as described above is only apparent in the upper region of the style, but not in the lower region.

## 4 Discussion

### 4.1 Towards a mechanistic model for pollination-dependent *S-RNase* expression in *Pyrus*

In this study, we examined the effect of pollination compatibility type on the allele-specific *S-RNase* expression in the upper and lower region of the style in the *P. communis* cultivar “Conference.” We found that in unpollinated styles *S-RNase* expression peaks at the balloon stage and gradually decreases after anthesis. A gradual decrease in “basal” *S-RNase* expression after anthesis was also observed in previous studies in *S. chacoense* (O’Brien et al., 2002) and *P. bretschneideri* (Shi et al., 2017), corroborating our findings. We also uncovered that this “baseline” expression profile in unpollinated styles did not significantly differ between the upper and lower region of the style. This is in line with a previous study in apple which found homogenous *S-RNase* expression along the entire length of the transmitting tract (Certal et al., 1999), but not with measurements in *S. chacoense* where elevated *S-RNase* expression was observed in the upper half of the style (O’Brien et al., 2002).

The results of our expression analysis also showed that the temporal *S-RNase* expression profile of both alleles, namely, *PcS*_*10*8_ and *PcS*_*121*_, is highly similar for each individual pollination treatment. The fact that both alleles follow the same expression pattern, even upon semi-compatible pollination, suggests that any signal mediating feedback regulation of the *S-RNase* expression during the occurrence of a pollen-pistil (in) compatibility reaction does not operate in an allele-specific manner but instead acts on both *S-RNase* alleles. However, we did notice that *Pc*S_108_ expression was consistently higher compared to *Pc*S_121_ for all pollination/no pollination treatments.

**FIGURE 6:**
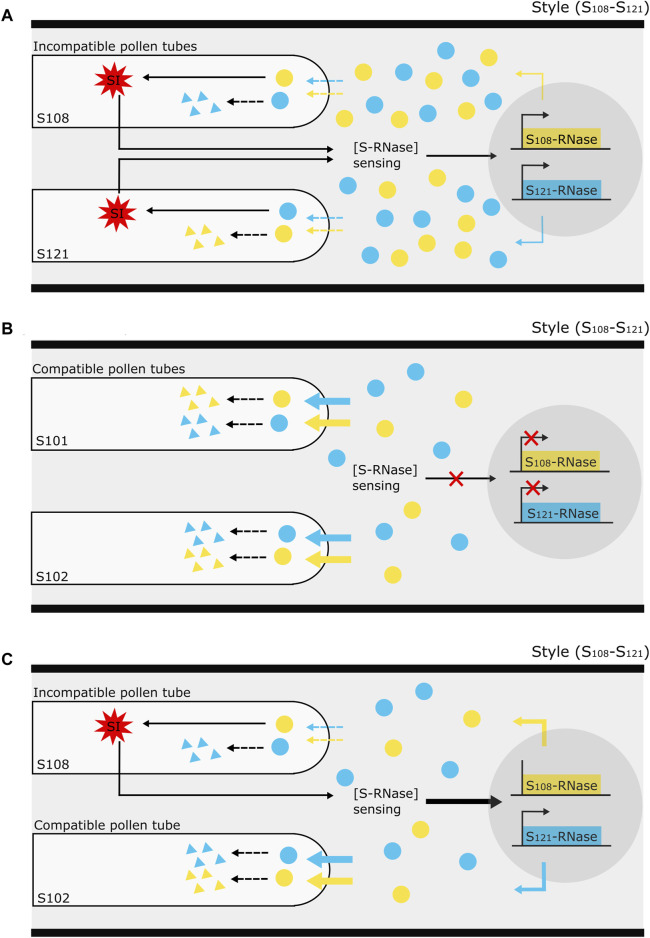
Illustration of the proposed mechanism underlying the effect of pollen tube (in)compatibility on *S-RNase* gene expression in the style of *Pyrus*. **(A)** The occurrence of an incompatibility reaction (red, multi-pointed star) in “self-type” pollen tubes sends a signal to the style to sense the stylar S-RNase protein concentration [S-RNase] and keep it at a minimum level required to maintain the self-incompatibility reaction. This minimum threshold amount is maintained by the *de novo* synthesis of extra S-RNase through a yet unknown system of transcriptional activation. **(B)** On the other hand, in the complete absence of an incompatibility reaction, such as upon cross-pollination, there is no activation of this [S-RNase] sensing mechanism to maintain S-RNase protein levels in the style, so that there is no feedback regulation on S-RNase expression and it thus follows the gradually decreasing “baseline” dynamics as occurs in the absence of pollination. Without active S-RNase replenishment from transcriptional activation, the continuous S-RNase degradation inside the compatible pollen tubes causes a general decrease in the stylar S-RNase protein level. **(C)** In the specific case of a semi-compatible pollination, the occurrence of an incompatibility reaction in the incompatible pollen tubes signals the style to sense the stylar S-RNase protein concentration. In the meantime, the progressive S-RNase degradation by the presence of compatible pollen tubes may strongly decrease S-RNase protein levels in the style therefore promoting S-RNase expression even further. The assumption that the depletion of non-self-S-RNase by incompatible pollen tubes is much lower than by compatible pollen tubes is schematically illustrated by dotted and full, colored arrows signifying the entrance of S-RNase protein into the pollen tubes.

The temporal *S-RNase* expression profiles of the unpollinated and cross-pollinated styles show a decreasing trend, whereas the expression profile after self-pollination shows constant expression. After semi-compatible pollination, the *S-RNase* expression in the upper region of the style also remained stable, though significantly higher compared to the other pollination types, followed by a delayed drop in expression at 3 dpp. Decreased *S-RNase* expression after cross-pollination and maintained expression after self-pollination have previously also been described in *S. chacoense* (O’Brien et al., 2002; Liu et al., 2009), lemon (Zhang et al., 2015; Li et al., 2022), and *P. bretschneideri* (Shi et al., 2017). Drawing upon these findings together with our own results in *P. communis* cv. “Conference,” we put forth a tentative hypothesis to explain variations in *S-RNase* allele expression patterns in pear, as influenced by the compatibility type of the pollination (Figure 6).

First, the model presumes that the *S-RNase* expression in the pistil gradually decreases in the absence of any pollination event. The occurrence of an incompatibility reaction from “self-type” pollen tubes in the style serves as a signal to maintain a minimum level of S-RNase protein, i.e., by promoting the production of extra S-RNase via transcriptional activation. As such, this mechanism guarantees sustained *S-RNase* expression following an incompatible pollination event (Figure 6A). In the case of a fully compatible pollination (“Cross”), no incompatibility reaction occurs in the style and the stylar S-RNase protein concentration [S-RNase] sensing mechanism is not activated. As a consequence, transcriptional promotion of the *S-RNase* gene is not activated, and expression of both *S-RNase* alleles decreases (Figure 6B) similar to when no pollination event has taken place. In the case of semi-compatible pollination, the incompatibility reaction in the “self-type” pollen tubes will signal the style to keep a constant S-RNase level. However, because of the parallel presence of compatible pollen tubes in the style, there is a progressive depletion of the stylar S-RNase pool (i.e., by diffusion and degradation into the cross-type pollen tubes), triggering an enhanced activation of *S-RNase* gene expression (Figure 6C). As such, the model not only explains the maintained *S-RNase* expression level upon selfing but also accounts for the enhanced expression of both *S-RNase* alleles upon semi-compatible pollination events.

One major assumption in the proposed mechanistic model is that the depletion of non-self-S-RNases by incompatible pollen tubes is much lower than in the metabolically healthy compatible pollen tubes, and therefore not or only minimally affects the overall [S-RNase] pool in the pistil, hence only having low to moderate activation effects on *S-RNase* transcription. This would explain why, in the semi-compatible treatment, enhanced *S-RNase* expression is higher than upon selfing (see Figures 3, 4), and why expression dynamics of *Pc*S_121_, which is degraded by both compatible and the incompatible pollen tubes, is highly similar to that of the *Pc*S_108_ allele, which is solely degraded by the compatible pollen tubes.

In the semi-compatible pollination treatment, differences in the temporal *S-RNase* expression pattern between the upper and lower part of the style were noted. This phenomenon may be explained by the presumption that the presence of incompatible pollen tubes activates the signaling to maintain *S-RNase* expression in the whole style, while the depletion of the S-RNase protein pool by the compatible pollen tubes enhances this signal in a spatial manner across the length of the style to hence confer a more pronounced increase in *S-RNase* expression in the upper as compared to the lower region of the style. The observed drop in *S-RNase* expression at 3 dpp after a semi-compatible pollination can be explained by the loss of the incompatibility reaction and downstream signaling activation due to the death of incompatible pollen tubes. The fact that this drop in *S-RNase* expression at 3 dpp is not seen after self-pollination may be due to the fact that, in our experiment, many more incompatible pollen tubes are present after a full selfing and therefore the incompatibility reaction and related downstream signaling is much stronger and more pronounced. In addition, in a full incompatibility reaction many more pollen tubes must be inhibited before the promotive effect on *S-RNase* expression is seized, expectantly leading to a prolonged promotive effect on *S-RNase* expression compared to a semi-compatible pollination.

### 4.2 Validation of the putative model in the context of previous research

The mechanism described here is not the first model that has been proposed to explain differential *S-RNase* expression patterns and protein levels in the pistil in response to pollination compatibility type. Liu et al. (2009) suggested a positive feedback mechanism based on their observations in wild potato stating that *S-RNase* mRNA levels in the style decreased much faster after cross-pollination compared to self-pollination. They hypothesized that the compatible pollen tubes during their growth through the style send out a signal that reduces *S-RNase* expression, thereby lowering S-RNase production and reinforcing the compatible reaction. However, in this case, one would expect an increased reduction of *S-RNase* expression after cross-pollination compared to no pollination, which did not corroborate with our results. Interestingly, an earlier study on the same species found very similar effects of the pollination compatibility type on stylar *S-RNase* expression as Liu et al. (2009), however, a completely different mechanistic model was put forward to explain this “feedback” regulation (O’Brien et al., 2002). These authors suggested that the presence of dead pollen tubes or molecules liberated from the arrested pollen tubes either directly or indirectly stimulate *S-RNase* gene transcription, or, suppress their mRNA turnover, to ensure sufficient amounts of S-RNase protein in the style to reject putatively new incoming pollen grains from incompatible genotypes (O’Brien et al., 2002).

Our model shares the presumption that incompatible pollen tubes (or the induction of their death) form a trigger to promote transcriptional activation of both *S-RNases* present in the style. However, we include the additional notion that the incompatibility reaction triggers the style to maintain a minimum level of S-RNase protein, i.e., with the actual level of S-RNase enzyme in the style additionally regulating the extent of *S-RNase* gene expression. This presumption is supported by pollination observations in earlier research. Liu et al. (2009) found that in *S. chacoense* S-RNase protein levels stayed constant after self- and no pollination, but dropped significantly after semi-compatible pollination, and even more upon cross-pollination, which is in line with our proposed model. Another study in *S. chacoense* reported that the style requires a minimum level of S-RNase enzyme to maintain the ability to reject incompatible pollen (Qin et al., 2006), which was in agreement with the threshold hypothesis formulated earlier in Petunia (Clark et al., 1990). As part of this threshold model, it has also been suggested that style-to-style and genotype-to-genotype variations in stylar S-RNase levels could explain pseudo-compatibility due to the observed weakening of the SI response in case of reduced levels of S-RNase in the style (Qin et al., 2001; 2006). In *S. chacoense* this critical threshold was set at 80 ng/style or 0.06 mg mL^−1^ stylar extract (Qin et al., 2006). Interestingly, in Japanese pear, a minimum S-RNase protein level to ensure complete self-incompatibility was estimated at the same order of magnitude, namely, 0.1 mg mL^−1^ stylar extract (Hiratsuka and Zhang, 2002; Qin et al., 2006). This supports the assumption in our proposed model that there is a minimum S-RNase protein level in the style that is necessary to maintain the self-rejection capacity of the style and which is maintained in the presence of incompatible pollen tubes.

Our proposed model basically involves a minimum threshold-based feedback regulation of *S-RNase* gene expression by the stylar [S-RNase] pool upon the occurrence of self-type pollination and therefore presumes the existence of a signaling mechanism that promotes *S-RNase* expression under conditions of self-incompatibility. An important question that remains is what this signal is or how this signaling would be achieved. One possibility is that the style registers the presence of (in) compatible pollen tubes via physical or biochemical changes in the transmitting tract. For example, compatible pollen tubes may be recognized through the uptake of stylar nutrients, while the presence of ROS or other metabolites that are the result of programmed cell death (PCD) might signal the presence of incompatible pollen tubes. In *Pyrus pyrifolia*, S-RNase action has been associated with depolymerization of the actin cytoskeleton, mitochondrial alteration and DNA degradation in incompatible pollen tubes (“self-type”), suggesting induction of programmed cell death (PCD) (Wang et al., 2009). A transcriptome analysis in lemon comparing stylar gene expression after selfing and cross-compatible pollination revealed that genes involved in calcium-mediated signaling, and NADPH oxidase, which plays a role in ROS signaling, were upregulated following self-pollination (Zhang et al., 2015). This study also showed evidence for the involvement of phytohormone signaling transduction pathways in the SI reaction and regulation of *S-RNase* expression. In line with this, comparative transcriptomics between self- and cross-pollinated styles in different plant species with S-RNase-dependent GSI, including *Pyrus bretschneirderi*, unveil differential expression of genes involved in phytohormone synthesis and signaling, including ethylene, auxin, jasmonate, cytokinin, and gibberellins (Zhao et aal, 2015; Shi et al., 2017). Among these plant hormones, jasmonate (JA) emerges as a promising candidate for orchestrating self-incompatibility regulation. Various observations have hinted at its participation in the self-incompatibility processes as elaborated below. However, while the actual contribution of this phytohormone remains formally unproven, it’s important to note that other metabolites could also potentially play a role. For example, in *P. bretschneideri* the concentration of JA in the style decreases significantly upon cross-pollination, but not upon self-pollination. Moreover, a key enzyme for JA biosynthesis, allene oxide cyclase (AOC), and the JA-associated MYC transcription factor, involved in the activation of JA-mediated systemic response to wounding, were found to be significantly downregulated in cross-pollinated but not in self-pollinated styles. Further supporting the functional involvement of jasmonic acid (JA) in *Pyrus* gametophytic self-incompatibility (GSI), it was observed that the external application of JA-Me significantly boosted the expression of *S-RNase* in the style. This observation implies a potential connection between diminished *S-RNase* expression in compatible styles and JA signaling (Shi et al., 2017). A similar transcriptome experiment on tomato styles also revealed significant upregulation of *JAZ* and *MYC2*, as well as significantly enriched plant hormone signal transduction-related KEGG pathway, after an incompatible pollen interaction, but not after cross-pollination (Zhao et al., 2015). On the other hand, a study in apple uncovered that JA is also involved in the regulation of self-incompatibility modifiers in the pollen tubes. Gu et al. (2019) found that the uptake of S-RNase promotes the accumulation of JA in pollen tubes, which stimulates the expression of *MdMYC2* and its target, a defensin gene, *MdD1*. This defensin gene inhibits the activity of both self- and non-self S-RNases by targeting the active site of the ribonuclease during the initial phases of pollen tube growth prior to the occurrence of self/non-self-recognition. The presumption that, in the style, *S-RNase* expression may be regulated by JA signaling while, in the pollen tube, S-RNase accumulation induces JA signaling seems contradictory. However, it may hint at the presence of multiple complex signaling networks that are activated independently in the style and pollen tube upon pollination and/or self-recognition. In order to get more insight into the feedback mechanism(s) that control *S-RNase* gene expression in *Pyrus* and thus regulate GSI, further research on the signaling events that occur after pollination and in response to self-incompatibility might benefit from using novel techniques that allow analysis of pollen tube and style events separately, like the analysis of male/female specific expression in the stigma upon pollination performed in *Arabidopsis thaliana* by Kodera et al. (2021). These insights will allow the validation of the proposed mechanistic model and will help to further unravel the intricate signaling network occurring at the style-pollen tube interface to determine the outcome of a pollination event in function of the compatibility of the parental genotypes.

## 5 Conclusion

Several previous studies have reported a differential effect of compatible and incompatible pollination on *S-RNase* expression in plant species that carry the S-RNase-dependent GSI system. In this study, allele-specific *S-RNase* expression analysis using RT-qPCR was performed in the pear cv. “Conference” in response to different types of pollination compatibility. Based on these results and previously reported findings, we here propose a model to explain dynamics of stylar *S-RNase* expression in response to pollination and compatibility type of growing pollen tubes. The model proposes that upon occurrence of an incompatibility reaction the style receives a signal to maintain a minimum critical threshold level of S-RNase protein by actively promoting its expression. In the absence of an incompatibility reaction, this signaling pathway is not activated and *S-RNase* expression drops gradually such as under non-pollinated conditions. More work is needed to unravel the actual mechanistic players of the proposed model.

## Data Availability

The original contributions presented in the study are included in the article/Supplementary Material, further inquiries can be directed to the corresponding author.

## Supplementary material

SUPPLEMENTARY FIGURE S1 Agarose gel showing the results of the S-genotyping PCR using consensus primers PyComC1F and PyComC5R (Sanzol, 2009). Electrophoresis was conducted on a 1.5% agarose gel stained with ethidium bromide. The GeneRuler Express DNA ladder was used to estimate the size of the genomic DNA fragments. Letters above the lanes denote the ID numbers of the different Pyrus cultivars used in this study: **(A)** “Légipont,” **(B)** “Conference,” **(C)** “Bartlett.” PCR bands of interest are marked by a red box.

SUPPLEMENTARY FIGURE S2 Nucleotide alignment of *Pc*S_101_ of cultivar “Bartlett” compared to the reference *Pc*S_101_ sequence (accession no. AB236428.1).

SUPPLEMENTARY FIGURE S3 Nucleotide alignment of *Pc*S_102_ of cultivar “Bartlett” and “Légipont” compared to the reference *Pc*S_102_ sequence (accession no. AB236425).

SUPPLEMENTARY FIGURE S4 Nucleotide alignment of *Pc*S_108_ of cultivar “Conference” and “Légipont” compared to the reference *Pc*S_108_ sequence (accession no. AB236427).

SUPPLEMENTARY FIGURE S5 Nucleotide alignment of *Pc*S_121_ of cultivar “Conference” compared to the reference *Pc*S_121_ sequence (accession no. AB236426).
