# Supplementary material for: The influence of the pollination compatibility type on the pistil S-RNase expression in European pear (Pyrus communis)
Source: Front Genet. 2024 Apr 9;15:1360332. doi: 10.3389/fgene.2024.1360332 (PMC11035772; doi:10.3389/fgene.2024.1360332)
Supplement: Supplementary file 1 [file Table1.docx]

**Supplementary Table 1:** List of primers used to determine the S-genotypes of “Conference” (PcS_108_-PcS_121_), “Légipont” (PcS_102_-PcS_108_), and “Bartlett” (PcS_101_-PcS_102_).

The table includes the primer names, primer type (consensus, specific, qPCR), the target gene/S-allele, the primer orientation, primer sequence, the primer-specific annealing temperature (Ta (°C)), and whether the primer pair was previously described in literature or designed specifically for this study.

| **Primer Name** | **Primer Type** | **Target** | **Orientation** | **Sequence** | **Ta (°C)** | **Source** |
| --- | --- | --- | --- | --- | --- | --- |
| PyComC1F | Consensus | Multiple | Forward | ATTTTCAATTTACGCAGCAATATCAGC | 54 | (Sanzol, 2009a) |
| PyComC5R | Consensus | Multiple | Reverse | CTG CAA AGW SHG ACC TCA ACC AAT TC | 54 | (Sanzol, 2009a) |
| FTC282 | Specific Primer | S101 | Forward | CCCAGATGATCACCTAAGGGC | 57 | This study |
| FTC283 | Specific Primer | S101 | Reverse | ACTGGCTTAGATGAGGGCAT |  | This study |
| FTC284 | Specific Primer | S101 | Forward | TGGATTGGGGAAGTTCACAC | 58 | This study |
| FTC285 | Specific Primer | S101 | Reverse | ACTCACACACACACTAACTAGC |  | This study |
| FTC286 | Specific Primer | S101 | Forward | AGCCTATAACCCATCGTATCGT | 59 | This study |
| FTC287 | Specific Primer | S101 | Reverse | TCCAACTGGGGTTTGAGTGA |  | This study |
| FTC288 | Specific Primer | S102 | Forward | CAGGTATACACGTCATCAGGGA | 59 | This study |
| FTC289 | Specific Primer | S102 | Reverse | TGCAGTTTTCTGGGTCAGGT |  | This study |
| FTC290 | Specific Primer | S102 | Forward | TTCAGGTACGCTTGTGCAAAT | 59 | This study |
| FTC291 | Specific Primer | S102 | Reverse | AGCTAGCCGCGCTCTTAATAAT |  | This study |
| FTC0212 | Specific Primer | S102 | Forward | CTGCCTGCAAGTTTCACCAT | 57 | This study |
| FTC0213 | Specific Primer | S102 | Reverse | ATCAACGAAAAAGAATGTACCCAT |  | This study |
| FTC292 | Specific Primer | S102 | Forward | AGGGTTTTAATACTCACACTT | 53 | This study |
| FTC293 | Specific Primer | S102 | Reverse | TGGGGTGGTATATATTAATGAG |  | This study |
| FTC0027 | Specific Primer | S102 | Forward | ACTGTCATGCAAGGGCTCAA | 58 | This study |
| FTC0028 | Specific Primer | S102 | Reverse | CGTGTCCTGCCCTTACCATC |  | This study |
| FTC316 | Specific Primer | S108 | Forward | AAGGGCATTGCACATGACTA | 55 | This study |
| FTC317 | Specific Primer | S108 | Reverse | TCTGGGTCAGGTCCCTTCTT |  | This study |
| FTC318 | Specific Primer | S108 | Forward | GGCGATGATTCAACCGAACG | 60 | This study |
| FTC319 | Specific Primer | S108 | Reverse | GCTAGCCAGCCTCGCTCT |  | This study |
| PyComS8F | Specific Primer | S108 | Forward | CTTGTAACGATCGTCCTGAACAA | 60 | (Sanzol, 2009a) |
| PyComS8R | Specific Primer | S108 | Reverse | CCTCAACTAATTCAGTCGTCGTC |  | (Sanzol, 2009a) |
| FTC0155 | Specific Primer | Multiple | Forward | CAC WTA GAA ACA CAA CAT ATG AAC AAA | 56 | This study |
| B53S21R2 | Specific Primer | S121 | Reverse | TTTGGTTTCTTATTGTTGATGCTC |  | (Sanzol, 2009a) |
| B52S21F2 | Specific Primer | S121 | Forward | TCACCCAGAAAATTGCACGGAC | 45 | (Sanzol, 2009a) |
| FTC0156 | Specific Primer | Multiple | Reverse | MTAKCYKCGCTCTTAAT |  | This study |
| B52S21F2 | Specific Primer | S121 | Forward | TCACCCAGAAAATTGCACGGAC | 60 | (Sanzol, 2009a) |
| B53S21R2 | Specific Primer | S121 | Reverse | TTTGGTTTCTTATTGTTGATGCTC |  | (Sanzol, 2009a) |
| FTC363 | qPCR | S108 | Forward | AGAAGGGACCTGACCCAGAA | 60 | This study |
| FTC364 | qPCR | S108 | Reverse | TGAGCCACTCTCTTTCCCAG |  | This study |
| FTC365 | qPCR | S121 | Forward | AAATTGCACGAACGCAACCG | 60 | This study |
| FTC366 | qPCR | S121 | Reverse | GGGATTCCCACAGCTGCC |  | This study |
| EF1F | qPCR | EF1a | Forward | GGTGTGAAGCAGATGATTTG | 60 | Liu et al. 2018 |
| EF1R | qPCR | EF1a | Reverse | TCACCCTCAAACCCAGATAT |  | Liu et al. 2018 |
| HistF | qPCR | Histidine | Forward | GTCAAGAAGCCCCACAGATAC | 60 | Liu et al. 2018 |
| HistR | qPCR | Histidine | Reverse | CTGGAAACGCAGATCAGTCTTG |  | Liu et al. 2018 |
