# Supplementary figures and images for: The influence of the pollination compatibility type on the pistil S-RNase expression in European pear (Pyrus communis)
Source: Front Genet. 2024 Apr 9;15:1360332. doi: 10.3389/fgene.2024.1360332 (PMC11035772; doi:10.3389/fgene.2024.1360332)

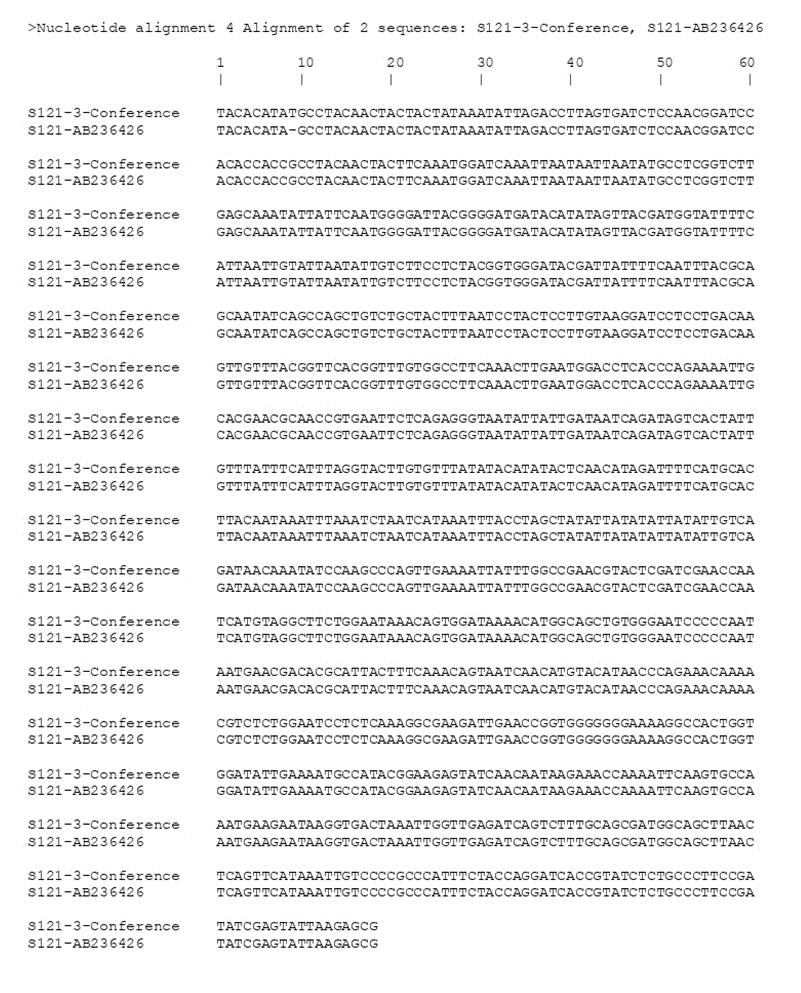

Supplement: Supplementary file 2 [file Image5.PNG]

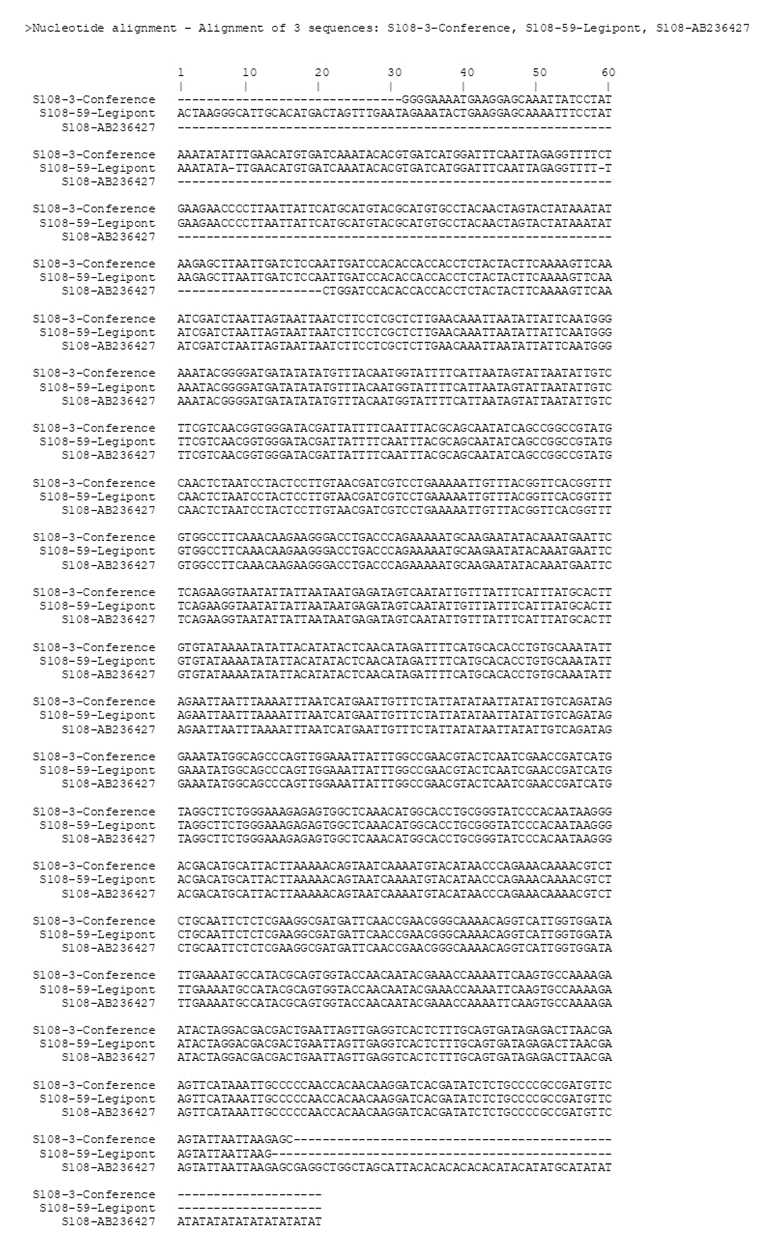

Supplement: Supplementary file 3 [file Image4.PNG]

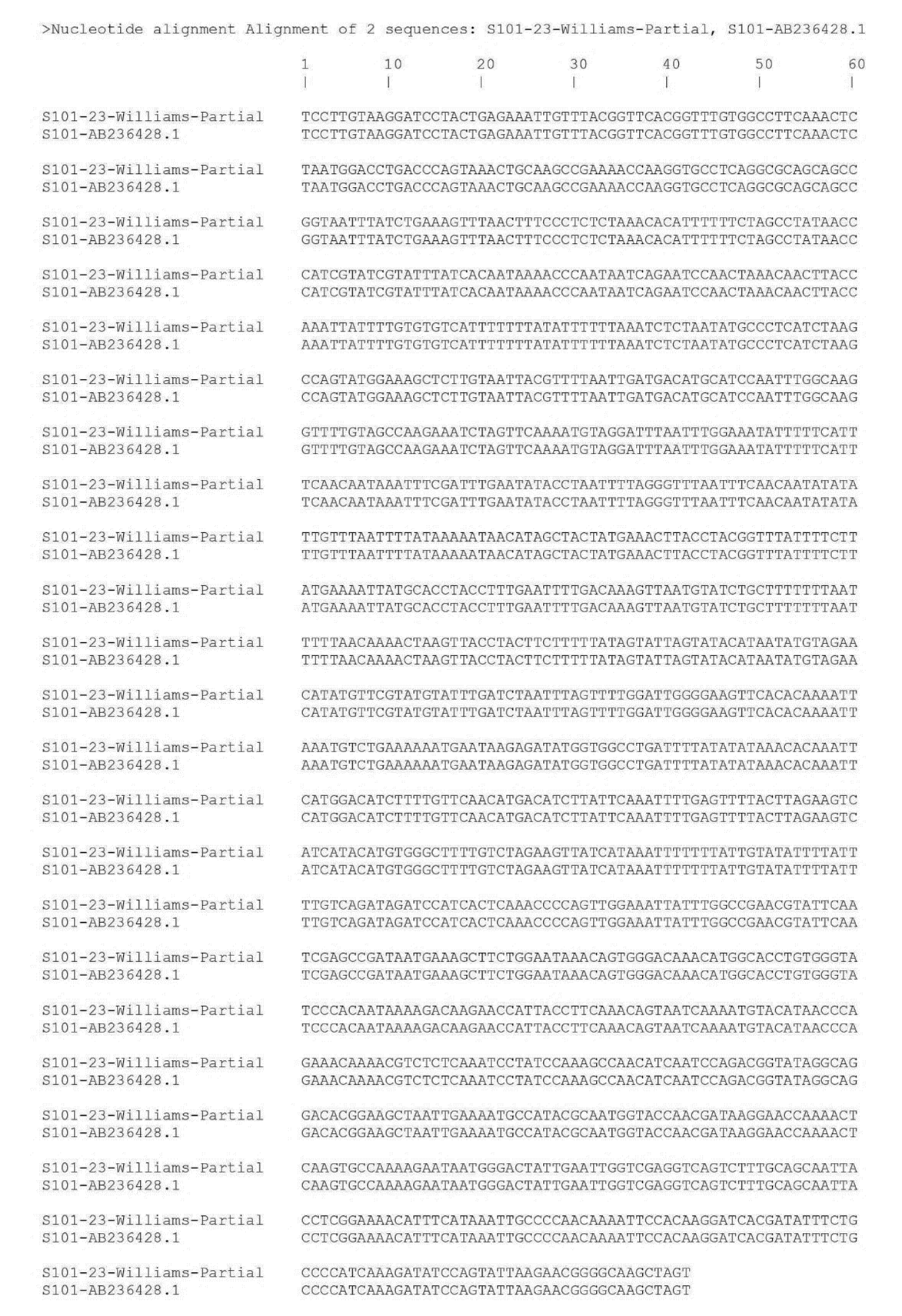

Supplement: Supplementary file 4 [file Image2.PNG]

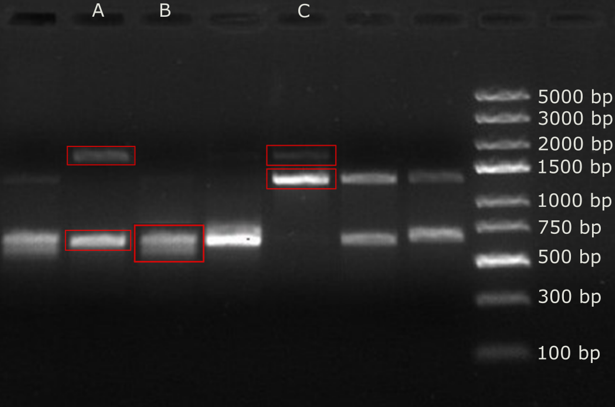

Supplement: Supplementary file 5 [file Image1.PNG]

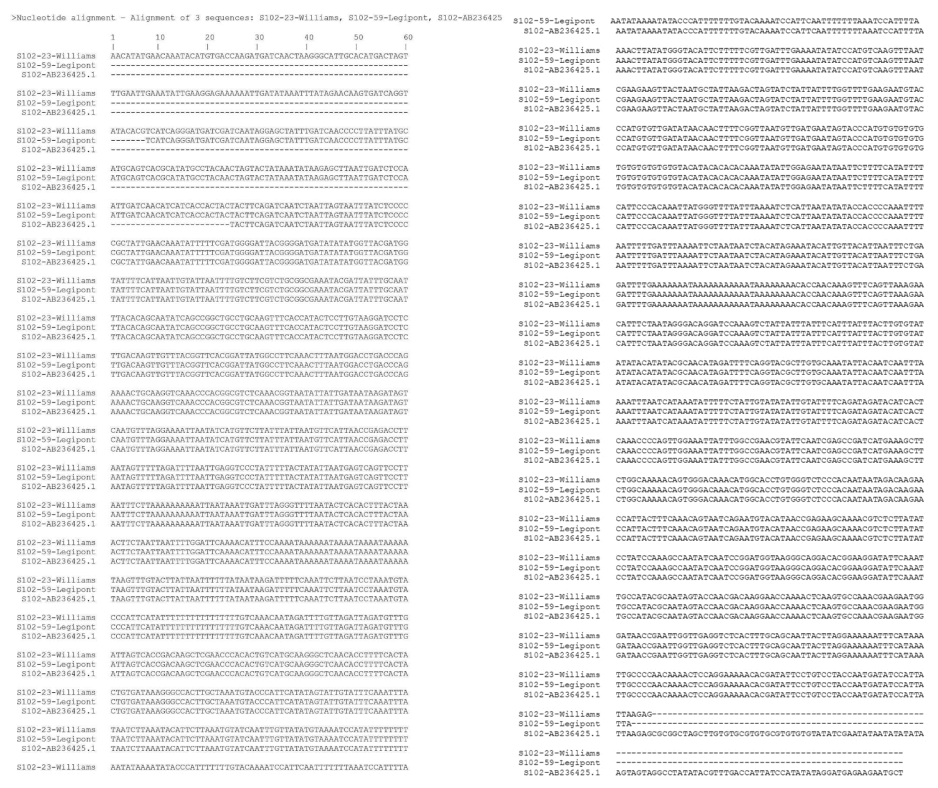

Supplement: Supplementary file 6 [file Image3.PNG]
